# Serum 25‐Hydroxyvitamin D3 Status in Adolescents With Anxiety Disorders: A Case‐Control and Vitamin D Supplementation Substudy

**DOI:** 10.1002/brb3.71095

**Published:** 2025-11-29

**Authors:** Wan Linfang, Liu Yang

**Affiliations:** ^1^ Pediatrics Department Jingzhou First People's Hospital Jingzhou Hubei Province China

**Keywords:** 25‐Hydroxyvitamin D_3_, adolescents, anxiety disorders, pediatric population

## Abstract

**Objective:**

To investigate the correlation between serum 25‐hydroxyvitamin D3 (25‐(OH)D3) levels and anxiety disorders in adolescents, and to assess the potential impact of vitamin D status on pediatric anxiety disorders, thereby providing clinical evidence for diagnosis and treatment.

**Methods:**

This study included 124 adolescents with anxiety disorders who visited the pediatric outpatient department of Jingzhou First People's Hospital from January 2020 to December 2022 as the experimental group and 131 healthy adolescents who underwent physical examinations during the same period as the control group. Serum 25‐(OH)D3 levels were compared between the two groups. Simple linear regression analysis was conducted to evaluate the predictive capacity of 25‐(OH)D3 levels on anxiety scores in the experimental group. Additionally, 86 adolescents with anxiety disorders and 25‐(OH)D deficiency or insufficiency in the experimental group received vitamin D supplementation for 12 weeks, and changes in anxiety scores before and after supplementation were analyzed to assess the effect of 25‐(OH)D3 levels on anxiety disorders.

**Results:**

The mean serum 25‐(OH)D3 level in the experimental group [(16.48 ± 6.53) ng/ml] was significantly lower than that in the control group [(22.95 ± 7.25) ng/ml], with a statistically significant difference (*p* < 0.05). The prevalence of vitamin D deficiency and insufficiency was higher in the experimental group (69.35%) than in the control group (36.64%), with a statistically significant difference (*p* < 0.05). Furthermore, in the experimental group, anxiety scores were negatively correlated with serum 25‐(OH)D_3_ levels, indicating that lower 25‐(OH)D_3_ levels were associated with higher anxiety scores. After vitamin D supplementation, adolescents with anxiety disorders and 25‐(OH)D deficiency or insufficiency exhibited reduced anxiety scores.

**Conclusion:**

Vitamin D deficiency may represent a potential risk factor for anxiety disorders in adolescents and demonstrate some predictive value for these conditions. Vitamin D supplementation could potentially alleviate anxiety symptoms in adolescents.

## Graphical abstract

Baseline characteristics of the study cohort, including adolescents with anxiety disorders (Experimental Group, *n* = 124) and age‐matched healthy controls (Control Group, *n* = 131). Age is presented as mean ± standard deviation; gender distribution is shown as frequency counts (percentage). An independent samples *t*‐test was used for age comparison and Pearson's chi‐square test for gender distribution. No statistically significant intergroup differences were observed in age (*t* = 1.156, *P* = 0.249, Cohen's *d* = 0.21) or gender (*χ^2^* = 0.000, *P* = 0.988, Cramer's *V* = 0.01).

Serum 25‐hydroxyvitamin D3 (25(OH)D) levels and deficiency categories in the experimental group (anxiety disorders, *n* = 124) versus healthy controls (*n* = 131). Deficiency is defined as 25(OH)D <12 ng/ml; insufficiency, 12–20 ng/ml; sufficiency, ≥20 ng/ml.

(A) Categorical distribution: The anxiety group showed significantly higher deficiency rates (25.0% vs. 6.1%) and lower sufficiency rates (30.6% vs. 63.4%) than controls (*χ^2^* = 27.340, df = 2, *P* < 0.001; Cramer's *V* = 0.327).

(B) Mean concentration: Serum 25(OH)D was markedly lower in the anxiety group (16.48 ± 6.53 ng/ml) versus controls (22.95 ± 7.25 ng/ml; *t* = −7.465, df = 253, *P* < 0.001; Cohen's *d* = −0.936, 95% CI: −1.19 to −0.68).

Distribution of serum 25‐hydroxyvitamin D (25(OH)D) levels by age subgroups within the anxiety disorder cohort (*n* = 124). Vitamin D categories: deficiency (<12 ng/ml), insufficiency (12–20 ng/ml), and sufficiency (≥20 ng/ml).

Categorical distribution: No significant differences in deficiency/insufficiency/sufficiency rates across age groups (*χ^2^* = 8.50, df = 6, *P* = 0.204; Cramer's *V* = 0.185).

Continuous levels: Median 25(OH)D concentrations (with interquartile range) showed no statistically significant variation among age subgroups (Kruskal–Wallis *H* = 0.962, df = 3, *P* = 0.810, epsilon squared = 0.008).

The prevalence of vitamin D deficiency ranged from 0% in the 15–16‐year‐old group to 12.5% in the ≥17‐year‐old group, while insufficiency was highest in the 15–16‐year‐old group (43.8%). Sufficiency was most common in the ≥17‐year‐old group (87.5%).

Median serum 25(OH)D levels were similar in the 11–12‐ and 13–14‐year‐old groups (23.5 ng/mL and 23.0 ng/mL, respectively), lower in the 15–16‐year‐old group (20.9 ng/mL), and highest in the ≥17‐year‐old group (29.4 ng/mL).

The chi‐square test indicated no statistically significant association between age group and vitamin D status categories, *χ^2^* (6) = 8.645, *p* = 0.195‐, Cramer's *V* = 0.182‐. Similarly, the Kruskal–Wallis test showed no significant difference in median 25(OH)D concentrations across age groups, H(3) = 4.50‐, *p* = 0.212‐, with a small effect size (*ε^2^* = 0.035).

Linear regression models examining the reciprocal relationship in adolescents with anxiety disorders (*n* = 124): Vitamin D → Anxiety: Serum 25(OH)D significantly predicted anxiety scores (B = −0.29, 95% CI: −0.34 to −0.23; *β* = −0.680, *t* = −10.24, *P* < 0.001), explaining 45.8% of variance (Adj. *R^2^* = 0.458). Anxiety → Vitamin D: Higher anxiety scores predicted lower 25(OH)D levels (*B* = −1.61, 95% CI: −1.92 to −1.30; *β* = −0.680, *t* = −10.24, *P* < 0.001) with identical effect size (Adj. *R^2^* = 0.458).

Both models demonstrated an absence of multicollinearity (VIF = 1.00) and followed the equations:

Comparison of anxiety scores (median with interquartile range) among vitamin D subgroups within the anxiety disorder cohort (*n* = 124): Deficiency (<12 ng/ml; *n* = 31): Significantly higher anxiety scores (median = 35, IQR = 34‐36) vs. insufficiency group (Mann–Whitney *U* = 10.0, *P* < 0.001; Cliff's *δ* = −0.988, 95% CI: −1.000 to −0.967) and vs. sufficiency group (*U* = 185, *P* < 0.001; *δ* = −0.686, 95% CI: −0.830 to −0.542).

No significant difference between insufficiency (12–20 ng/ml; *n* = 55; median = 32, IQR = 30 − 33) and sufficiency groups (≥20 ng/ml; *n* = 38; *U* = 965.5, *P* = 0.529; *δ* = −0.076, 95% CI: −0.250 to 0.098).

*P*‐values adjusted by Bonferroni correction for multiple comparisons.

Comparison of anxiety scores (mean ± SD) before and after 12‐week vitamin D supplementation in adolescents with anxiety disorders:

Vitamin D deficient group (<20 ng/ml; *n* = 86): Significant reduction from 32.83 ± 2.47 to 21.36 ± 4.57 (paired *t*‐test, *P* < 0.001). Vitamin D sufficient group (≥20 ng/ml; *n* = 38): Significant reduction from 31.97 ± 3.15 to 24.37 ± 4.25 (*P* < 0.001).

Between‐group analysis showed:

→ No baseline difference (*P* = 0.107 by independent *t*‐test)

→ Greater improvement in deficient group post‐treatment (mean difference = −3.01 points; *P* = 0.001).

Changes in serum 25(OH)D levels (median with IQR) and anxiety scores (mean ± SD) pre‐ vs. post‐treatment in vitamin D deficient adolescents with anxiety disorders (*n* = 86): Vitamin D: increased from 13.65 ng/ml (IQR: 9.94−16.71) to 28.13 ng/ml (IQR: 24.97−32.08; Wilcoxon signed‐rank test, *P* < 0.001).

Anxiety scores: Decreased from 32.83 ± 2.47 to 21.36 ± 4.57 (paired *t*‐test, *P* < 0.01)

Post‐treatment correlation analysis revealed a strong negative association between 25(OH)D levels and anxiety scores (Pearson's *r* = −0.665, *P* < 0.001).

Linear regression models in vitamin D‐supplemented adolescents (*n* = 86) after 12‐week intervention: Vitamin D → Anxiety: Post‐treatment 25(OH)D levels strongly predicted residual anxiety symptoms (*B* = −0.58, 95% CI: −0.73 to −0.44; *β* = −0.665, *t* = −8.15, *P* < 0.001), explaining 43.5% of variance (Adj. *R^2^* = 0.435). Anxiety → Vitamin D: Residual anxiety predicted 25(OH)D concentrations (*B* = −0.76, 95% CI: −0.94 to −0.57; *β* = −0.665, *t* = −8.15, *P* < 0.001) with identical effect size (Adj. *R^2^* = 0.435).

Both models confirmed the absence of multicollinearity (VIF = 1.00).

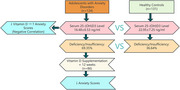

## 1 Introduction

Adolescent anxiety disorders are among the most prevalent psychological conditions during adolescence, characterized by excessive worry, anxiety, and fear. According to the Diagnostic and Statistical Manual of Mental Disorders (DSM‐5), these conditions fall under the diagnostic category of “Anxiety Disorders.” Existing research demonstrates that anxiety in children and adolescents can significantly affect their social adaptability, physical and mental health, and personality development (Kassa et al. 2024; Wang et al. 2024), with the adverse effects potentially worsening over time (Rapee et al. 2023). The onset of anxiety disorders during childhood and adolescence may hinder healthy development, a concern that has increasingly garnered attention from parents and healthcare professionals. As the most common psychiatric problems, anxiety disorders affect approximately 1 in 12 children and 1 in 4 adolescents (Kowalchuk et al. 2022; Liu et al. 2024). Another study further indicated that the prevalence rate of childhood anxiety disorders was 16.7% when functional impairment was not considered, while the clinical diagnosis prevalence decreased to 5.2% after accounting for functional impairment (Mutluer et al. 2023). Anxiety disorders encompass specific phobias, social anxiety disorder, separation anxiety disorder, agoraphobia, panic disorder, and generalized anxiety disorder (Kowalchuk et al. 2022). The pathogenesis and etiology of childhood anxiety disorders remain unclear, and they are currently considered by the academic community to result from the interplay of multiple factors—such as environmental influences, parenting styles, biological elements, and family history—with research suggesting potential associations with genetics (Weber et al. 2025), social environment and stress (Kowalchuk et al. 2022; Warner and Strawn 2023), family education approaches (Aaron and Kiel 2024; Kadoglou et al. 2024), and cognition (Warner and Strawn 2023; Chorot et al. 2023), among others. Current research indicates that structural impairments within the fronto‐temporo‐limbic pathway, functional dysconnectivity, and dyshomeostasis in neuroendocrine regulation collectively constitute the core pathological mechanisms underlying anxiety disorders (Shan et al. 2023; Shan et al. 2024; Chang et al. 2024; Dark et al. 2023; Bonaz et al. 2024; Chen et al. 2023; Borchers et al. 2024).

Treatment for adolescent anxiety disorders typically involves a multifaceted approach, including behavioral therapy (Ortiz and Fastman 2024), cognitive‐behavioral therapy (Stiede et al. 2023), pharmacotherapy (Mills et al. 2024), family support, and educational interventions (Smith and Monga 2025). Psychotherapy is generally regarded as the first‐line intervention, with Cognitive Behavioral Therapy (CBT) widely established as an effective treatment for mild to moderate anxiety (Stiede et al. 2023; Pettitt et al. 2022; Rapee et al. 2023). While pharmacotherapy is typically considered as an adjunct or alternative, particularly in cases of moderate severity (Zugman et al. 2024; Stiede et al. 2024). While medication has been shown to be effective for adolescence anxiety disorders (Mills et al. 2024), limitations exist regarding drug types (Nicotra and Strawn 2023), safety (Hampsey et al. 2023), long‐term effects (Kim et al. 2023), and potential dependency (Hampsey et al. 2023).

The pathogenesis of childhood anxiety disorders remains incompletely elucidated, and their clinical management continues to pose challenges against the backdrop of limited therapeutic options. In recent years, growing attention has been directed towards non‐psychopharmacological intervention strategies, with the relationship between vitamin D and mental health emerging as a research focus. Beyond its classical roles in calcium‐phosphate homeostasis and skeletal health, vitamin D is extensively involved in various physiological processes, including immunomodulation, oxidative stress response, hormone secretion, and neurodevelopment (Argano et al. 2025, Sailike et al. 2024). Its potential implications in cardiovascular diseases, metabolic disorders, and mental health issues are also increasingly being recognized (Naganuma et al. 2022; Kaneva et al. 2022).

In the neuropsychiatric domain, vitamin D is regarded as a neurosteroid, as its active metabolites can cross the blood‐brain barrier and be detected in the cerebrospinal fluid (Zajkowska et al. 2022). Research indicates that vitamin D can modulate the metabolism of key neurotransmitters such as dopamine and serotonin, thereby potentially influencing the pathogenesis and progression of anxiety (Kouba et al. 2024, Cui et al. 2025). Observational studies further suggest that vitamin D deficiency is associated with various psychiatric conditions, including major depressive disorder and anxiety disorders in adults (Wen et al. 2024), while the widespread insufficiency observed in children may correlate with an increased risk of neuropsychiatric diseases, including anxiety disorders (Kazemi et al. 2023; Muskens et al. 2022; Wang et al. 2023; Tokarchuk et al. 2022; Hatzimanolis et al. 2024; Zhou et al. 2024; Zhou et al. 2024; Marazziti et al. 2023; Wang et al. 2024).

However, evidence regarding the specific link between vitamin D and childhood anxiety disorders remains inconsistent. Some clinical studies indicate that low vitamin D levels may negatively impact anxiety symptoms in children (Wen et al. 2024; Wu et al. 2021), and vitamin D supplementation has shown potential for alleviating anxiety in certain trials (Wen et al. 2024; Borges‐Vieira and Cardoso 2023). Conversely, other studies have not confirmed a significant therapeutic effect (Renteria et al. 2024), and meta‐analyses indicate that, despite an inverse correlation with anxiety symptoms, evidence for the efficacy of vitamin D as a standalone intervention is insufficient (Zaromytidou et al., 2022). One large‐scale meta‐analysis found that daily supplementation with 1000 IU of vitamin D led to a slight improvement in depressive symptoms but did not significantly affect anxiety symptoms (Ghaemi et al. 2024).

This inconsistency may stem from interference by multiple confounding factors. Vitamin D levels are influenced by season, gender, geographic latitude, and metabolic complexity, including the presence of metabolites such as C3‐epi‐25‐hydroxyvitamin D_3_ (Chen et al. 2023; Chen et al. 2024; Zadro Matovina et al. 2023). Furthermore, variables like BMI, dietary intake, physical activity, and socioeconomic status are often inadequately controlled in studies, contributing to heterogeneous findings.

Therefore, although basic research supports the important role of vitamin D in neurodevelopment and emotional regulation, its clinical significance in childhood anxiety disorders and the efficacy of supplementation require further validation through more rigorously designed, well‐controlled, high‐quality studies.

This study aims to investigate the association between serum 25‐hydroxyvitamin D_3_ levels and anxiety scores in adolescents, thereby further elucidating the risk factors for adolescence anxiety disorders and providing a scientific foundation for identifying new treatment approaches.

The findings of this study will facilitate a deeper understanding of the correlation between adolescence anxiety disorders and vitamin D levels, offering novel insights into the auxiliary diagnosis and treatment of anxiety disorders in children.

## 2 Research Subjects and Methods

### 2.1 Subjects

This study adopted a retrospective design. The experimental group comprised 124 adolescents with anxiety disorders who visited the pediatric outpatient department of Jingzhou First People's Hospital for their first consultation. The control group consisted of 131 healthy adolescents who underwent routine physical examinations at our hospital during the same period. All participants were aged ≥11 years and <18 years, a developmental period classified as adolescence by the World Health Organization. Within pediatric research and ethical frameworks, this age group is also encompassed under the broader term “children.”

### 2.2 Ethical Review and Consent

This study was reviewed and approved by the Ethics Committee of Jingzhou First People's Hospital. All procedures adhered strictly to ethical guidelines. Prior to participation in the study, written informed consent forms were obtained from all parents or legal guardians of the adolescents involved. The personal information and health data of all participants were maintained with strict confidentiality.

### 2.3 Inclusion and Exclusion Criteria

All participants underwent diagnostic assessments conducted by licensed child psychiatrists certified by the Chinese Society of Psychiatry, using the following protocol:

#### 2.3.1 Diagnostic Verification

Anxiety disorders: Confirmed via Structured Clinical Interview for DSM‐5 Anxiety Disorders (SCID‐5‐AD) and ICD‐11 Clinical Descriptions and Diagnostic Guidelines (CDDG)

Symptom validation: ≥2 independent clinical evaluations (Cohen's κ = 0.86, 95% CI [0.79, 0.93])

#### 2.3.2 Exclusion Screening

(1) Mood disorders: Systematically screened with:
(2) Kiddie Schedule for Affective Disorders and Schizophrenia (K‐SADS‐PL)
(3) Children's Depression Inventory (CDI) score >65
(4) Young Mania Rating Scale (YMRS) score >12

#### 2.3.3 Other Exclusions

(1) Vitamin D supplementation
(2) Neurological/developmental disorders (EEG/MRI‐confirmed)
(3) Substance‐induced symptoms (excluded by participant‐reported non‐exposure history)
(4) ADHD (Conners 3 T‐score >65) / psychosis (Positive and Negative Syndrome Scale >60)

### 2.4 Research Methods

A total of 255 adolescents who underwent serum 25‐(OH)D_3_ testing at the Pediatric Psychiatry Clinic of Jingzhou First People's Hospital between January 2020 and December 2022 were enrolled in this study. All participants were independently assessed by two physicians using the Structured Clinical Interview for DSM‐5 Anxiety Disorders (SCID‐5‐AD) and the ICD‐11 Clinical Descriptions and Diagnostic Guidelines (CDDG) to confirm the presence or absence of anxiety disorders. Individuals with other mood disorders or meeting any exclusion criteria were excluded.

From the cohort, 124 adolescents diagnosed with anxiety disorders were assigned to the experimental group, and 131 healthy adolescents were included as the control group. Serum 25‐(OH)D_3_ levels were compared between the two groups. The adolescents with anxiety disorders were further stratified into four age groups at 2‐year intervals (24 months): 11–12 years (*n* = 28), 13–14 years (*n* = 54), 15–16 years (*n* = 32), and ≥17 years (*n* = 10). Differences in 25‐hydroxyvitamin D_3_ levels among these groups were analyzed.

Within the experimental group, anxiety scale scores were compared across vitamin D deficient, insufficient, and sufficient subgroups using the DSM‐5 Level 2—Anxiety—Child Age 11–17 scale. The control group did not undergo this assessment.

Among the participants, 86 adolescents with vitamin D deficiency or insufficiency received oral vitamin D supplementation combined with psychological and behavioral therapy, while those with sufficient vitamin D levels received psychological and behavioral therapy only. Anxiety scale scores were assessed before and after a 12‐week treatment period using the DSM‐5 Level 2—Anxiety—Child Age 11–17, and the relationship between vitamin D levels and anxiety scores was analyzed.

### 2.5 Anxiety Scale Scoring

Two senior pediatric psychiatrists administered the self‐reported DSM‐5 Level 2‐Anxiety‐Child Age 11‐17 scale using standardized protocols. Clinicians verbally presented all 13 items to participants in Mandarin Chinese, after which participants independently rated the frequency of their general anxiety symptoms over the prior week on a 5‐point Likert scale (1 = None to 5 = Always; total score range: 13–65) (PROMIS Health Organization and PROMIS Cooperative Group 2012e). Clinicians directly recorded responses on scoring sheets without interpretation. The validated Chinese version of this scale demonstrates excellent reliability (Cronbach's α = 0.90) (Youwen et al. 2018), with its self‐report nature capturing subjective symptom burden while clinician facilitation ensures comprehension.

### 2.6 25‐Hydroxyvitamin D Detection

The initial blood sample was collected following a pediatric outpatient visit and prior to any treatment intervention. A 3 mL peripheral venous blood sample was obtained from all participants—including both the anxiety disorder experimental group and the healthy physical examination control group—in the morning after an overnight fast. After 12 weeks of vitamin D supplementation, a second blood sample was collected under the same conditions (morning, fasting state) from only those adolescents who had received vitamin D supplementation. All samples were processed and analyzed in the same clinical laboratory (outpatient laboratory) using standardized protocols. Serum 25‐hydroxyvitamin D_3_ [25‐(OH)D] levels were measured using liquid chromatography‐tandem mass spectrometry (LC‐MS/MS).

Vitamin D status was classified according to the “Expert Consensus on the Clinical Application of Vitamin A and Vitamin D in Chinese Children” issued by the Child Health Care Branch of the Chinese Preventive Medicine Association (Chinese Society of Preventive Medicine. Child Health Branch 2024), as well as the 2016 Global Consensus Statement on the Management of Nutritional Rickets (Munns et al. 2016): 25‐(OH)D < 12 ng/mL indicated deficiency; 12 ng/mL ≤ 25‐(OH)D < 20 ng/mL indicated insufficiency; 20 ng/mL ≤ 25‐(OH)D ≤ 100 ng/mL indicated sufficiency; and 25‐(OH)D > 100 ng/mL indicated excess. Since 25‐(OH)D_3_ is the predominant circulating form of vitamin D, its measured value was used interchangeably with total 25‐(OH)D in this study. For simplicity, vitamin D deficiency refers to 25‐(OH)D deficiency, vitamin D insufficiency refers to 25‐(OH)D insufficiency, and low vitamin D status encompasses both deficiency and insufficiency. These terms will not be repeated in subsequent sections.

### 2.7 Treatment Methods

All adolescents in the experimental group received psychological and behavioral therapy guidance. Active involvement of parents and family‐based interventions was encouraged to support treatment adherence. Among these, 86 adolescents with vitamin D deficiency or insufficiency were identified and administered 400 IU of oral vitamin D once daily for 12 consecutive weeks.

### 2.8 Statistical Methods

Statistical analyses were performed using SPSS 27.0 (IBM Corp.). Continuous variables with normal distribution are expressed as mean ± standard deviation (SD), non‐normally distributed data as median (interquartile range), and categorical variables as frequencies (percentages). Normality was verified by Shapiro–Wilk tests (*p* > 0.05). Specific analyses included:

1. Intergroup comparisons (Experimental vs. Control)
  Independent t‐test:
  - Age difference
  - Serum 25(OH)D levels
  - Baseline anxiety scores

Chi‐square test:

- Gender distribution
- Vitamin D status proportions (deficient/insufficient/sufficient)

2. Subgroup analyses

Mann–Whitney *U* test: Vitamin D levels across age strata

Chi‐square test: Vitamin D category distribution by age

3. Association analysis

Pearson correlation: Serum 25(OH)D levels vs. anxiety scores

4. Within‐experimental group analyses

One‐way ANOVA with Tukey post hoc:

- Anxiety score differences among vitamin D status subgroups
- Significance threshold: *p* < 0.01 for ANOVA omnibus test

Paired t‐test: Anxiety score changes pre/post vitamin D supplementation

5. Effect size reporting

Cohen's *d* (*t*‐tests)

Phi coefficient (chi‐square)

Pearson's *r* (correlation)

Partial *η^2^* (Eta squared) (ANOVA)

Cliff's *δ* (Delta) (non‐parametric comparison)

*ε^2^* (Epsilon squared) (Kruskal–Wallis test)

*R^2^* (R‐squared) (regression)

*β* (Beta) (regression)

*B* (Unstandardized coefficient) (regression)

Cramer's *V* (chi‐square for >2 × 2 tables)

All tests were two‐tailed with primary significance set at *p* < 0.05, except ANOVA omnibus tests, which required *p* < 0.01 due to multiple comparisons.

## 3 Results

### 3.1 Comparison of General Data: Comparison of Gender Distribution and Age Characteristics Between Experimental and Control Groups

The experimental group comprised 124 newly diagnosed adolescents with anxiety disorders, with a mean age of 14.31 years (SD = 1.68), including 73 boys and 51 girls. The control group consisted of 131 healthy adolescents, with a mean age of 14.06 years (SD = 1.67), including 77 boys and 54 girls. Age comparison between groups showed a small effect size difference (Cohen's *d* = 0.21), though this difference was not statistically significant (*t* = 1.24, *p* = 0.217). The gender distribution exhibited a trivial effect size difference (Cramer's *V* = 0.01) with no significant difference between groups (*χ^2^* = 0.38, *p* = 0.537). These results indicate that the demographic characteristics of the two groups were well‐balanced, as detailed in Table 1.

**TABLE 1:** Demographic characteristics by Study Group.

Characteristic	Experimental Group (*n* = 124)	Control Group (*n* = 131)	Statistics	*p*‐value	Effect size
**Age, years**					
Mean ± SD	14.31 ± 1.68	13.92 ± 2.05	*t* = 1.156	0.249	Cohen's *d* = 0.21
**Gender**					
Male, *n* (%)	73 (58.9%)	77 (58.8%)	*χ^2^* = 0.000	0.988	Cramer's *V* = 0.01
Female, *n* (%)	51 (41.1%)	54 (41.2%)			

### 3.2 Comparison of Serum 25‐Hydroxyvitamin D Levels between the two Groups of Adolescents

Blood samples from both groups were collected in the morning after an overnight fast, with no statistically significant difference in collection conditions (*P* > 0.05). The serum 25‐hydroxyvitamin D level in the healthy control group was significantly higher than that in the anxiety disorder group (*t* = −7.465, *P* < 0.05, |*d*| = 0.936, indicating a large effect size). Furthermore, the prevalence of vitamin D deficiency and insufficiency was higher in the anxiety disorder group (69.35%) compared to the control group (36.64%), with a statistically significant difference. The distribution of vitamin D status differed significantly between the experimental and control groups (χ^2^ = 27.34, *p* < 0.001), with a medium effect size (Cramer's *V* = 0.327). See Table 2.

**TABLE 2:** Comparison of 25(OH)D levels/distribution between Experimental and Control Groups.

Group	*n*	Deficient *n* (%)	Insufficient *n* (%)	Sufficient *n* (%)	25(OH)D (ng/ml), Mean ± SD
Experimental	124	31 (25.0%)	55 (44.4%)	38 (30.6%)	16.48 ± 6.53
Control	131	8 (6.1%)	40 (30.5%)	83 (63.4%)	22.95 ± 7.25
Statistics		χ^2^ = 27.340, df = 2 Cramer's *V* = 0.327			*t* = −7.465, df = 253, Cohen's *d* = −0.936 (95% CI: −1.19 to −0.68)
*p*‐value		< 0.001			< 0.001

### 3.3 Comparison of Serum 25‐Hydroxyvitamin D Levels Among Adolescents Across Different Age Groups

The experimental group in this study included adolescents with anxiety disorders aged over 11 years. Based on the age distribution analysis, the majority of adolescents with anxiety disorders were concentrated in the 13‐14‐year‐old age group. Furthermore, an examination of vitamin D levels across different age groups revealed that the 13‐14‐year‐old group exhibited the most widespread vitamin D deficiency.

We stratified the participants by age into two‐year intervals (11–12, 13–14, 15–16, and ≥17 years) and compared the distribution of vitamin D levels across these groups to evaluate differences in vitamin D status among adolescents with anxiety disorders in different age groups. Given the relatively small sample sizes within each age group, we employed the rank‐sum test (Mann–Whitney *U* test). The statistical results demonstrated that at a significance level of *α* = 0.05, the p‐values for pairwise comparisons between groups were all substantially higher than 0.05, indicating no significant differences in vitamin D levels across the age groups. Additionally, we examined whether there were differences in the proportions of vitamin D deficiency, insufficiency, and sufficiency across the age groups using the chi‐square test. The results showed that the *p*‐value was greater than 0.05, indicating no statistically significant differences. Cramer's *V* = 0.185 indicates a weak association between age and vitamin D status in the experimental group. *ε^2^* = 0.008, indicating a trivial effect size. This suggests that age group accounted for only 0.8% of the variance in vitamin D levels, implying that age variation across the adolescent range (11–17 years) had no significant effect on vitamin D levels. See Table 3. We similarly analyzed the relationship between vitamin D levels and age distribution in the control group. The results indicated no statistically significant association between age groups and vitamin D status categories, x^2^(6) = 8.645, *p* = 0.195, Cramer′s *V* = 0.182. Similarly, the Kruskal Wallis test showed no significant difference in median 25 (OH)D concentrations across age groups, H(3) = 4.50, *p* = 0.212, with a small effect size (ε^2^ = 0.035) (see Table 4). Therefore, we conclude that vitamin D levels did not differ significantly across age groups in either the experimental or the control group.

**TABLE 3:** Vitamin D levels and age distribution in the Experimental Group.

Age group (years)	Total *n*	Deficient *n* (%)	Insufficient *n* (%)	Sufficient *n* (%)	25(OH)D (ng/ml), Median [IQR]
11–12	28	7 (25.0%)	14 (50.0%)	7 (25.0%)	16.4 [11.9, 21.1]
13–14	54	15 (27.8%)	21 (38.9%)	18 (33.3%)	18.0 [11.3, 21.5]
15–16	32	6 (18.8%)	18 (56.2%)	8 (25.0%)	14.0 [12.5, 20.8]
≥17	10	3 (30.0%)	2 (20.0%)	5 (50.0%)	18.2 [10.6, 22.7]
Statistics		*χ^2^* = 8.50, df = 6 *p* = 0.204 Cramer's *V* = 0.185			*H* = 0.962, df = 3, *P* = 0.810, *ε^2^* = 0.008

### 3.4 Analysis of the Relationship Between 25‐(OH)D_3_ Levels and Anxiety Scores in Adolescents With Anxiety Disorders

This analysis was performed within the experimental group. The cases of low serum 25‐hydroxyvitamin D_3_ (deficiency and insufficiency) were statistically evaluated. Specifically, there were 31 adolescents in the vitamin D deficiency group, 55 in the insufficiency group, and 38 in the sufficiency group. Descriptive statistics and correlation analysis were employed to assess the relationship between vitamin D levels and anxiety scores. *T*‐tests or one‐way ANOVA were used to compare anxiety scores across different vitamin D level groups. The results indicated a significant negative linear relationship between vitamin D levels and anxiety scores before treatment, with an ANOVA regression significance of less than 0.01 and a Pearson's correlation coefficient (r) of ‐0.68.

Bidirectional regression analysis was performed to assess the relationship between baseline vitamin D levels and anxiety scores in the experimental group. The results revealed a strong, symmetric association between vitamin D levels and anxiety scores (*β* = –0.680). Specifically. For every 1‐point increase in anxiety score, vitamin D levels decreased by 1.61 ng/ml (95% CI: –1.92 to –1.30, *P* < 0.001). Conversely, for every 1 ng/ml decrease in vitamin D level, anxiety scores increased by 0.29 points (95% CI: 0.23 to 0.34, *P* < 0.001). The explanatory power was identical in both directions (*R^2^* = 0.462), indicating that the two variables share 46.2% of their variance. See Table 5.

**TABLE 4:** Vitamin D levels and age distribution in the Control Group.

Age group (years)	Total *n*	Deficient *n* (%)	Insufficient *n* (%)	Sufficient *n* (%)	25(OH)D (ng/ml), Median [IQR]
11–12	35	3 (8.6%)	9 (25.7%)	23 (65.7%)	23.5 [17.6, 28.8]
13–14	56	4 (7.1%)	16 (28.6%)	36 (64.3%)	23.0 [16.5, 27.5]
15–16	32	0 (0%)	14 (43.8%)	18 (56.2%)	20.9 [18.4,27.5]
≥17	8	1 (12.5%)	0 (0%)	7 (87.5%)	29.4 [25.3, 33.3]
Statistics		*χ^2^* = 8.645, df = 6, *p* = 0.195 Cramer's *V* = 0.182			*H* = 4.50, df = 3, *P* = 0.212, *ε^2^* = 0.035

*Note*: *H* = Kruskal–Wallis *H* statistic.

In addition, the cases in the experimental group were categorized into three groups according to their pre‐treatment vitamin D levels: vitamin D deficiency, vitamin D insufficiency, and vitamin D sufficiency. The rank sum test was employed to compare the pre‐treatment anxiety scores among these three groups. At the significance level of *α* = 0.05, the p‐values for the comparisons between the vitamin D deficiency group and the vitamin D insufficiency group, as well as between the vitamin D deficiency group and the vitamin D sufficiency group, were both significantly below 0.05, indicating substantial differences in pre‐treatment anxiety scores. Conversely, the *p*‐value for the comparison between the vitamin D insufficiency group and the vitamin D sufficiency group exceeded 0.05, suggesting no significant difference in pre‐treatment anxiety scores between these two groups. See Table 6.

**TABLE 5:** Bidirectional regression analysis of vitamin D levels and anxiety scores.

Model direction	Predictor	*B* (95% CI)	*β*	*t*	*p*	*R^2^*	Adj. *R^2^*	VIF
VitD → Anxiety	Constant	37.32 (36.33–38.30)	—	74.97	< 0.001	0.462	0.458	—
	Vitamin D	−0.29 (−0.34 to −0.23)	−0.680	−10.24	< 0.001	—	—	1.00
Anxiety → VitD	Constant	68.87 (58.72–79.03)	—	13.42	< 0.001	0.462	0.458	—
	Anxiety score	−1.61 (−1.92to −1.30)	−0.680	−10.24	< 0.001	—	—	1.00

**Abbreviations**: *B* = unstandardized coefficient, *β* = standardized coefficient, CI = confidence interval, VIF = variance inflation factor.

Regression equations:.

Model 1: Anxiety = 37.32 − 0.29 × VitD. Model 2: VitD = 68.87 − 1.61 × Anxiety.

### 3.5 The Impact of Vitamin D Supplementation on Anxiety Scores and Therapeutic Efficacy in Adolescents With Anxiety Disorders and Low Vitamin D Levels

All adolescents in the anxiety group received psychological and behavioral therapy guidance, along with active engagement of parental education and family‐based therapeutic cooperation. In the experimental group, 86 adolescents with low vitamin D levels were additionally administered oral vitamin D supplementation for a duration of 12 weeks.

After 12 weeks of treatment, a statistical analysis was performed based on the changes in anxiety scores. Adolescents with an improvement in anxiety scores of ≤ 10 points were classified as showing no effect, those with an improvement between 10 and 16 points were classified as showing moderate effect, and those with an improvement of ≥ 16 points were classified as showing significant effect. The pre‐ and post‐treatment anxiety score comparison and statistical analysis for the anxiety group (n = 124) revealed that 63 cases (50.80%) showed no effect, 54 cases (43.55%) showed moderate effect, and 7 cases (5.65%) showed significant effect.

We observed that, compared to pre‐treatment levels, the anxiety scores of all adolescents decreased to varying extents post‐treatment. Consequently, additional statistical analyses were performed to further investigate these changes.

Statistical analysis was conducted on the anxiety scale scores of adolescents with low vitamin D levels (*n* = 86) and normal vitamin D levels (*n* = 38) in the anxiety group before and after treatment. The results demonstrated that, compared to pre‐treatment scores, the anxiety scores of adolescents with anxiety disorders significantly decreased post‐treatment, with a statistically significant difference (*p* < 0.05). No significant difference was observed in anxiety scores between the two groups prior to treatment. However, a statistically significant difference emerged in anxiety scores between the two groups after treatment (*p* = 0.001 < 0.01), with the post‐treatment anxiety scores of the low vitamin D group being significantly lower than those of the sufficient vitamin D group. These findings suggest that vitamin D supplementation in adolescents with anxiety disorders and low vitamin D levels may alleviate their anxiety symptoms. See Table 7.

**TABLE 6:** Comparison of anxiety scores by vitamin D status in the Experimental Group.

Comparison	*n*_1_	*n*_2_	Median1 (IQR)	Median2 (IQR)	*U*	*P*	Adj *P*	Cliff's δ[95% CI]
Deficiency vs. Insufficiency	31	55	35 (34, 36)	32 (30, 33)	10.0	< 0.001	< 0.001	0.9883 [0.967, 1.000]
Insufficiency vs. Sufficiency	55	38	32 (30, 33)	32 (30, 33.25)	965.5	0.529	1.000	−0.0761[−0.250, 0.098]
Sufficiency vs. Deficiency	38	31	32 (30, 33.25)	35 (34, 36)	185	< 0.001	< 0.001	−0.6859[−0.830, −0.542]

*Note*: *U* = Mann–Whitney *U* statistic.

We conducted statistical analyses on the changes in vitamin D levels before and after treatment, as well as the corresponding post‐treatment anxiety scores, for adolescents with low vitamin D levels. The Pearson correlation coefficient between 25‐(OH)D_3_ (12 W) and anxiety score (12 W) in the low vitamin D group was −0.665, indicating a moderate negative correlation between these two variables. In other words, as the level of 25‐(OH)D_3_ (12 W) increases, the anxiety score (12 W) tends to decrease. These findings suggest that post‐treatment vitamin D levels may be a factor influencing post‐treatment anxiety scores. Improving low vitamin D levels could potentially aid in the treatment of anxiety disorders in adolescents. See Table 8.

**TABLE 7:** Comparison of anxiety scores before and after treatment across different vitamin D levels within the Experimental Group.

Group	*n*	Anxiety score before treatment (points)	Anxiety score after treatment (points)	*p*‐value (within‐group)
Vitamin D deficient	86	32.83 ± 2.47	21.36 ± 4.57	< 0.001
Vitamin D sufficient	38	31.97 ± 3.15	24.37 ± 4.25	< 0.001
Between‐group *p*‐value		0.107	0.001	

Following vitamin D supplementation in the experimental group (*n* = 86), bidirectional regression analyses were performed to re‐examine the relationship between vitamin D levels and anxiety scores. Post‐intervention analyses revealed:

Model 1 (Vitamin D predicting anxiety): For every 1‐unit increase in vitamin D level, anxiety scores decreased by 0.58 units. The regression slope significantly increased in magnitude from −0.29 (pre‐intervention) to −0.58 (post‐intervention).
Model 2 (Anxiety predicting vitamin D): For every 1‐unit increase in anxiety score, vitamin D levels decreased by 0.76 units. The regression slope decreased in magnitude from −1.61 (pre‐intervention) to −0.76 (post‐intervention).

Importantly, the standardized regression coefficient (β) showed only a negligible change, from −0.680 (pre‐intervention) to −0.665 (post‐intervention). This stability in β indicates that the strength of the inverse association between vitamin D levels and anxiety scores remained consistent before and after the intervention. See Table 9.

**TABLE 8:** Changes in vitamin D, anxiety scores, and their post‐treatment correlation in the vitamin D Supplementation Group (*n* = 86).

Parameter	Before treatment	After treatment	*p*‐value
25(OH)D (ng/mL)			
Median (Q1, Q3)	13.65 (9.94, 16.71)	28.13 (24.97, 32.08)	< 0.001
Anxiety Score			
Mean ± SD	32.83 ± 2.47	21.36 ± 4.57	< 0.01
Correlation analysis (Post‐Treatment)			
Pearson's *r* (25(OH)D vs. Anxiety)	—	—	−0.665

**TABLE 9:** Bidirectional regression analysis of vitamin D levels and anxiety scores after vitamin D Supplementation.

Model direction	Predictor	*B* (95% CI)	*β*	*t*	*p*	*R^2^*	Adj. *R^2^*	VIF
VitD → Anxiety	Constant	38.10 (33.92, 42.28)	—	18.13	< 0.001	0.442	0.435	—
	Vitamin D	−0.58 (−0.73, −0.44)	−0.665	−8.15	< 0.001	—	—	1.00
Anxiety → VitD	Constant	44.98 (40.96, 48.99)	—	22.26	< 0.001	0.442	0.435	—
	Anxiety score	−0.76 (−0.94,−0.57)	−0.665	−8.15	<0.001	—	—	1.00

**Abbreviations;** *B* = unstandardized coefficient, *β* = standardized coefficient, CI = confidence interval. Regression equations:

Model 1: Anxiety = 38.10 − 0.58 × VitD.

Model 2: VitD = 44.98 − 0.76 × Anxiety.

## 4 Discussion

This case‐control study with interventional follow‐up demonstrates a significant association between serum 25‐(OH)D_3_ levels and childhood anxiety disorders. The key findings include: (1) Children with anxiety disorders had significantly lower serum 25‐(OH)D_3_ levels than the healthy controls; (2) the prevalence of vitamin D insufficiency and deficiency was markedly higher in the anxiety group; (3) anxiety scores were inversely correlated with serum 25‐(OH)D_3_ levels among affected children; and (4) supplementation in children with vitamin D insufficiency/deficiency was associated with an alleviation of anxiety symptoms.

These findings are consistent with several observational studies reporting an inverse relationship between vitamin D status and anxiety symptoms in children and adolescents (Wen et al. 2024; Wu et al. 2021). However, our conclusion that vitamin D supplementation can alleviate anxiety is not entirely consistent with some studies, particularly certain meta‐analyses (Renteria et al. 2024; Zaromytidou et al., 2022; Ghaemi et al. 2024). This discrepancy may stem from the following key reasons:

1. Study population and baseline differences. The intervention in this study specifically targeted anxiety‐diagnosed children with confirmed vitamin D insufficiency/deficiency. This suggests that the potential benefit of supplementation may be highly dependent on baseline nutritional status and might not be observable in populations with normal vitamin D levels. Many trials reporting negative results did not stratify participants based on baseline vitamin D levels, potentially diluting the overall effect.
2. Synergistic effect of combined intervention. In the present study's subgroup, all participants with vitamin D insufficiency/deficiency received standard psychobehavioral therapy concurrently with vitamin D supplementation. The observed improvement may therefore stem from a synergistic interaction between nutritional correction and psychological treatment. By addressing an underlying biological deficit, vitamin D supplementation might enhance the patient's responsiveness to psychotherapy. This context fundamentally differs from studies investigating vitamin D as a standalone intervention.

Vitamin D deficiency is a global concern, with documented reports across various countries. In China, studies have demonstrated that vitamin D deficiency and insufficiency are prevalent among healthy children aged 0 to 18 years (Anna et al. 2021). Specifically, the severe deficiency rate is 2.46% (ranging from 1.03% to 4.47%), while the overall deficiency rate is 21.57% (ranging from 13.65% to 30.72%). Notably, the highest prevalence of 56.14% (ranging from 39.54% to 72.07%) occurs during adolescence (Chinese Society of Preventive Medicine, Child Health Branch 2024). Additionally, in China, the detection rate of vitamin D deficiency and insufficiency among children under 7 years old is 14%, with this rate gradually increasing with age (Luanluan et al. 2022). In the present study, 131 healthy children were included, of whom 48 exhibited vitamin D deficiency or insufficiency, accounting for 36.64%. This finding aligns closely with existing epidemiological data.

Children with anxiety disorders appear to have a higher prevalence of vitamin D deficiency compared to non‐anxious peers, with preliminary observations suggesting this metabolic alteration may be particularly prevalent in the adolescent subgroup (aged 11‐18 years) within this clinical population. In this study, the proportion of adolescents with insufficient or deficient vitamin D levels in the experimental group was significantly higher than that in the control group, suggesting that low vitamin D levels may be associated with an increased risk of anxiety disorders. Existing clinical evidence indicates that vitamin D deficiency may elevate the incidence of various conditions, including autism spectrum disorders, anxiety and depression, and schizophrenia (Horsdal et al. 2025). Furthermore, insufficient vitamin D levels may increase the likelihood of cognitive, social, and emotional developmental issues in individuals (Shekhawat et al. 2025). An increasing body of research highlights that patients with anxiety disorders tend to have lower vitamin D levels compared to healthy controls (Wen et al. 2024; Arabshahi et al. 2024).

In this study, the term “vitamin D insufficiency or deficiency” is provisionally referred to as “low vitamin D status.” The findings indicate that adolescents with anxiety disorders exhibited significantly lower serum 25‐(OH)D_3_ levels compared to healthy adolescents. Within the experimental group, adolescents with vitamin D deficiency demonstrated markedly higher anxiety scores than those with vitamin D insufficiency or sufficiency. These results suggest that low vitamin D status may contribute to the development of anxiety disorders in adolescents and that vitamin D deficiency could represent a potential risk factor for such disorders. Furthermore, the assessment of vitamin D levels may serve as an auxiliary diagnostic tool for children with anxiety disorders, providing valuable guidance for clinical diagnosis.

Age‐stratified analyses indicated no significant influence of age (11–17 years) on vitamin D levels among adolescents. Notably: (1) Despite lacking statistical significance, a numerical trend was observed: the lowest median level (14.0 ng/mL) occurred in the 15‐16‐year group, while the highest median level (18.2 ng/mL) was found in the ≥17‐year group; (2) Substantial variability was evident, with all groups exhibiting interquartile ranges (IQR) spanning approximately 7–8 ng/mL (e.g., 15‐16‐year group: 12.5–20.8 ng/mL). Potential reasons for vitamin D deficiency in adolescents may involve insufficient supplementation, rapid pubertal growth, or reduced outdoor activity (Baranoglu Kilinc and Bolu 2023). Additionally, endocrine changes during adolescence and heightened academic pressure may serve as contributing factors to the development of anxiety disorders.

Bidirectional regression analysis within the experimental group suggested a potential bidirectional vicious cycle between vitamin D deficiency (VDD) and anxiety symptoms. A low vitamin D status may exacerbate anxiety through specific biological mechanisms, while anxiety‐related behaviors (e.g., reduced outdoor activity) can further diminish vitamin D synthesis, creating a positive feedback loop.

Analysis of anxiety scores across different vitamin D statuses within the experimental group revealed that VDD was associated with significantly higher anxiety scores compared to sufficient status. Crucially, our data indicate that vitamin D levels may need to fall into the “deficient” range (<20 ng/mL, with a pronounced effect observed below 12 ng/mL in our cohort) to significantly impact anxiety. This aligns with neurobiological evidence demonstrating vitamin D's role in regulating serotonin (5‐HT) synthesis; VDD may disrupt the balance of anxiety‐related neurotransmitters [Relevant Citation Needed—replace with actual citation].

Based on these findings, we hypothesize that a serum vitamin D concentration below 12 ng/mL might represent a critical threshold for contributing to anxiety disorders. We recommend incorporating vitamin D screening into the routine assessment of individuals with anxiety disorders. Targeted supplementation should be considered for those identified as deficient. Future intervention studies are warranted to validate the therapeutic potential of vitamin D supplementation in managing anxiety.

Animal experiments have demonstrated that vitamin D treatment may improve anxiety symptoms in rats (Al‐Ramadhan et al. 2023). Additionally, studies on patients with anxiety disorders have shown that vitamin D supplementation positively influences the reduction of anxiety scores (Ignacio‐Mejía et al. 2025). This study confirmed the anxiolytic potential of vitamin D supplementation. Anxiety scores in adolescents with anxiety disorders who received vitamin D supplementation were significantly improved compared to pre‐treatment levels, and this improvement was markedly greater than in adolescents with anxiety disorders who did not receive vitamin D supplementation. Causal attribution of anxiety score reductions exclusively to vitamin D supplementation is precluded by co‐administered behavioral therapy and the absence of a vitamin D monotherapy control arm. Consequently, the additional benefit observed in the group with a low vitamin D status may be attributed to a confluence of factors rather than a sole effect of vitamin D supplementation. Despite these limitations, our findings suggest that providing supplementation to individuals with a low vitamin D status may hold additive value when combined with standard psychobehavioral counseling. In summary, this study indicates that vitamin D supplementation for individuals with a low vitamin D status could be a promising adjunct strategy to foundational psychobehavioral therapy. However, given the constraints inherent in the non‐randomized design, a definitive causal relationship remains unclear. Final conclusions await verification from future, rigorously designed randomized controlled trials.

The selection of an appropriate vitamin D supplementation dosage initially posed a challenge. Several guidelines, such as those from the Endocrine Society, recommend 400–600 IU/day as the lower limit for maintenance dosing in adolescents, particularly for non‐obese individuals with mild underlying health conditions (Tarikere Satyanarayana et al. 2023; Deruyter et al. 2023). Although no direct studies have addressed the suitability of vitamin D supplementation for anxiety disorders, some research suggests that higher doses (e.g., 1200 IU/day) may be necessary to alleviate adult anxiety symptoms (Ghaemi et al. 2024). Other studies indicate that even lower doses of vitamin D (e.g., 400 IU/day) can significantly increase serum 25(OH)D levels in vitamin D–insufficient individuals, which may be attributed to rapid absorption and metabolic sensitivity under deficient conditions.

In individuals with vitamin D insufficiency (serum total 25(OH)D <20 ng/mL), serum 25(OH)D concentrations rise rapidly within the first 4 weeks after initiating vitamin D supplementation (Shan et al. 2025). This may be related to low baseline vitamin D–binding protein (VDBP) saturation in a deficient state, facilitating the accumulation of free 25(OH)D. Furthermore, one study reported that, over the same intervention period, a dose of 2000 IU/day resulted in an approximately two‐fold greater increase in 25(OH)D levels compared to a 400 IU/day regimen, though the latter still led to a statistically significant elevation (Bowman et al. 2024). In terms of long‐term cumulative effects, supplementation for 12 weeks (approximately three months) covers more than the half‐life of vitamin D (2–3 weeks), allowing blood concentrations to reach a steady state (Oortgiesen et al. 2023).

While the potential beneficial effect of low‐dose vitamin D on adolescent anxiety disorders observed in this study is encouraging, certain limitations should be noted. Serum 25(OH)D levels were not systematically measured at multiple timepoints before and after supplementation, and adherence was assessed solely based on reports from parents and participants, which may have introduced reporting bias.

This study conducted bidirectional regression analyses to examine the relationship between vitamin D levels and anxiety scores at two time points: before and after the intervention (vitamin D supplementation combined with behavioral therapy). The observed changes in regression slopes across models suggest that the intervention may have amplified the role of vitamin D in reducing anxiety, particularly among adolescents with low baseline vitamin D levels. Post‐intervention elevations in vitamin D levels potentially reflect a partial buffering of the negative impact of anxiety on vitamin D status.

Crucially, the *β* coefficients for the vitamin D‐anxiety association demonstrated minimal change across the pre‐ and post‐intervention bidirectional regression analyses (from −0.680 to −0.665). This stability in the strength of the negative association supports the potential utility of vitamin D as a biomarker for anxiety. Furthermore, the data suggest that changes in vitamin D levels exert a more pronounced influence on anxiety within the lower range of vitamin D concentrations. By elevating vitamin D levels, the intervention may have reduced the “consumptive” effect of anxiety on vitamin D—potentially mediated by anxiety‐related behaviors (e.g., reduced sunlight exposure)—or mitigated its behavioral impact.

Consequently, vitamin D supplementation appears to be particularly beneficial for anxious adolescents with vitamin D insufficiency/deficiency, demonstrating enhanced efficacy in reducing anxiety symptoms post‐intervention. Collectively, these findings indicate that maintaining sufficient vitamin D levels may confer bidirectional benefits for mental health: directly contributing to anxiety reduction while simultaneously diminishing the negative feedback loop where anxiety exacerbates vitamin D depletion. For adolescents with low vitamin D, targeted supplementation represents a viable adjunctive strategy for anxiety management, especially given the observed post‐intervention enhancement of its effect.

In conclusion, there is likely a significant association between vitamin D levels and the occurrence of anxiety disorders in children. Preliminary evidence tentatively suggests that vitamin D supplementation might yield modest improvements in anxiety symptoms among vitamin D‐insufficient adolescents with anxiety disorders. The findings of this study underscore the critical importance of evaluating vitamin D levels in the diagnosis and treatment of adolescent anxiety disorders and offer a promising new direction for further research into the therapeutic management of anxiety disorders in children.

This study has several limitations that warrant acknowledgment. First, the non‐randomized, open‐label design and the absence of a placebo‐controlled arm for the supplementation cohort preclude definitive causal inferences. Crucially, all participants received psychobehavioral counseling, while only those with a low vitamin D status received supplementation. This entanglement of interventions means the observed improvements cannot be solely attributed to vitamin D and may be confounded by the non‐specific effects of additional clinical attention. Second, the 36‐month study duration encompassed substantial seasonal variation in sunlight exposure, a key determinant of 25(OH)D_3_ levels; however, the sampling strategy did not account for or model these seasonal/monthly fluctuations. Furthermore, the analysis did not adjust for several potential confounders, including BMI/obesity, dietary intake, physical activity, socioeconomic status, medication use, comorbidities, or geographic latitude, which substantially limits the internal validity of the findings. Third, the assessment of anxiety severity relied on clinician‐supervised self‐report scales, which are susceptible to response biases (e.g., social desirability, recall) and may lack diagnostic rigor. Fourth, medication adherence was not objectively quantified (e.g., via pill counts), introducing uncertainty regarding actual supplement exposure. Finally, the single‐center design and modest sample size limit the generalizability of our findings to broader pediatric populations.

## Author Contributions

L. Y. conceptualized the study and revised the manuscript. W. L. conducted the experiments and collected data and performed statistical analysis. All authors reviewed and approved the final manuscript.

## Funding

This study was funded by the Science and Technology Research Project of the Education Department of Hubei Province (Grant No. B2023026).

## Conflicts of Interest

The authors declare no conflicts of interest.

## Data Availability

The datasets generated and/or analyzed during this study are not publicly available to protect patient privacy and comply with ethical regulations approved by the Ethics Committee of Jingzhou First People's Hospital (Approval No.: KY202438). De‐identified data may be shared upon reasonable request to the first author at dnve_007@163.com, contingent on approval by the ethics committee and the establishment of a formal data‐sharing agreement.
